# Sexual exploitation, abuse and harassment in humanitarian contexts

**DOI:** 10.2471/BLT.24.291655

**Published:** 2024-09-25

**Authors:** Jasmine-Kim Westendorf, Junru Bian, Megan Daigle, Alina Potts, Kathleen Jennings, Moira Reddick, Carl Cecil Massonneau, Gaya Gamhewage, Mohamed Esam Mahmoud

**Affiliations:** a Department of Politics, Media and Philosophy, La Trobe University, Kingsbury Drive, Bundoora 3086, Victoria, Australia.; b School of Political Studies, University of Ottawa, Ottawa, Canada.; c ODI, London, England.; d The Global Women’s Institute, George Washington University, Washington DC, United States of America.; e Department of Sociology and Political Science, Norwegian University of Science and Technology, Trondheim, Norway.; f Reproductive Health and Research, World Health Organization, Geneva, Switzerland.; g Prevention and Response to Sexual Misconduct, Office of the Director-General, World Health Organization, Geneva, Switzerland.

## Abstract

Considerable investment has been made in recent years to address sexual exploitation, abuse and harassment by aid workers in the humanitarian sector. However, such sexual misconduct remains a persistent, complex challenge with wide-ranging impacts, including on sexual health, for individuals and communities hosting humanitarian responses. This article considers the state of research regarding sexual exploitation, abuse and harassment in humanitarian contexts, and identifies gaps in the evidence base necessary for reinforcing prevention and response efforts. We first report what we know about sexual exploitation, abuse and harassment, including its impacts on sexual health, risk factors and the permissive enabling organizational cultures. We then identify several critical knowledge gaps that must be addressed for more effective future strategies and approaches to prevent and respond to sexual exploitation, abuse and harassment. We discuss system-wide knowledge gaps, such as lack of evidence on programming approaches and effectiveness of prevention and accountability mechanisms. We explore potential options that health-care programming provides for preventing and responding to sexual exploitation, abuse and harassment. We also describe population-level knowledge gaps, including in patterns of perpetration and specific challenges faced by marginalized groups. We conclude with reflections for a future integrated research and policy agenda.

## Résumé

Des investissements considérables ont été réalisés ces dernières années pour lutter contre le harcèlement, les abus et l'exploitation sexuelle perpétrés par des travailleurs du secteur de l'aide humanitaire. Toutefois, une telle inconduite sexuelle demeure un problème complexe et chronique aux multiples répercussions, notamment sur la santé sexuelle, pour les individus et communautés recevant une assistance humanitaire. Le présent article s'intéresse à l'état des recherches relatives au harcèlement, aux abus et à l'exploitation sexuelle dans des contextes humanitaires, et identifie les lacunes dans les bases factuelles nécessaires pour renforcer les efforts de prévention et d'intervention. Nous commençons par exposer ce que nous savons au sujet de cette problématique, y compris ses répercussions sur la santé sexuelle, les facteurs de risque et les cultures organisationnelles permissives. Ensuite, nous recensons les nombreuses lacunes majeures en termes de connaissances, qu'il faut impérativement combler pour que les futures stratégies et approches soient plus efficaces en matière de prévention et de lutte contre le harcèlement, les abus et l'exploitation sexuelle. Nous constatons que ces lacunes sont également présentes dans l'ensemble du système, se traduisant par exemple par l'absence de données factuelles sur la planification des approches et sur l'efficacité des mécanismes de prévention et de responsabilisation. Par ailleurs, nous examinons les différentes options que proposent les programmes de soins de santé pour se prémunir contre le harcèlement, les abus et l'exploitation sexuelle, mais aussi y remédier. Nous évoquons également le manque d'informations au sein de la population, notamment dans les schémas d'exécution et les difficultés spécifiques rencontrées par les groupes marginalisés. Nous concluons par une série de réflexions sur un futur programme intégré mêlant recherches et décisions politiques.

## Resumen

En los últimos años, se ha invertido mucho para abordar la explotación, el abuso y el acoso sexuales por parte de los trabajadores humanitarios del sector. Sin embargo, estas conductas sexuales inapropiadas siguen siendo un problema persistente y complejo, con repercusiones de gran alcance, incluso en la salud sexual, para las personas y las comunidades que acogen las respuestas humanitarias. Este artículo analiza el estado de la investigación sobre la explotación, el abuso y el acoso sexuales en los contextos humanitarios, e identifica las deficiencias en la base empírica necesaria para reforzar las iniciativas de prevención y respuesta. En primer lugar, se informa sobre lo que se conoce acerca de la explotación, el abuso y el acoso sexuales, incluidas sus repercusiones en la salud sexual, los factores de riesgo y las culturas organizativas permisivas. Luego, se identifican varias deficiencias críticas en el ámbito de los conocimientos que deben abordarse para que las estrategias y los enfoques futuros sean más eficientes en la prevención y la respuesta a la explotación, el abuso y el acoso sexuales. Se discuten las deficiencias de conocimiento en todo el sistema, como la falta de evidencia sobre los enfoques de programación y la eficiencia de los mecanismos de prevención y rendición de cuentas. También se exploran las posibles opciones que ofrece el sistema sanitario para prevenir y responder a la explotación, el abuso y el acoso sexuales. Asimismo, se describen las deficiencias de conocimiento a nivel poblacional, incluidas las pautas de perpetración y los desafíos específicos a los que se enfrentan los grupos marginados. Por último, se presentan algunas reflexiones para una futura agenda integrada de investigación y política.

## ملخص

لقد تم وضع استثمار كبير خلال السنوات الأخيرة في التعامل مع الاستغلال الجنسي والإساءة والتحرش، والصادر عن العاملين في مجال المساعدات الإنسانية في القطاع الإنساني. ومع ذلك، فإن سوء السلوك الجنسي هذا لا يزال يشكل تحديًا مستمرًا ومعقدًا، وله تأثيرات واسعة النطاق، بما في ذلك تأثيرات على الصحة الجنسية، بالنسبة للأفراد والمجتمعات التي تستضيف الاستجابات الإنسانية. تتناول هذه المقالة حالة البحث المتعلق بالاستغلال الجنسي والإساءة والتحرش في الأوضاع الإنسانية، وتحدد الفجوات في قاعدة الأدلة اللازمة لتعزيز جهود الوقاية والاستجابة. نقوم أولاً بتوضيح ما نعرفه عن الاستغلال الجنسي والإساءة والتحرش، بما في ذلك تأثيراتها على الصحة الجنسية، وعوامل الخطر، وثقافات المؤسسات المسموح بها والتي تدعم ذلك. ونحدد بعد ذلك العديد من الفجوات المعرفية الحرجة التي يجب التعامل معها من أجل استراتيجيات وأساليب مستقبلية أكثر فعالية لمكافحة الاستغلال الجنسي والإساءة والتحرش، والاستجابة لها. نحن نناقش الفجوات المعرفية على مستوى النظام، مثل الافتقار إلى الأدلة حول مناهج البرمجة وفعالية آليات المكافحة والمساءلة. ونستكشف أيضًا الخيارات المحتملة التي توفرها برمجة الرعاية الصحية لمكافحة الاستغلال الجنسي والإساءة والتحرش، والاستجابة لها. كما نصف هنا فجوات المعرفة على مستوى السكان، بما في ذلك أنماط ارتكاب هذه التعديات، والتحديات المحددة التي تواجهها الفئات المهمشة. ونختتم بأفكار لأجندة بحثية وسياسية متكاملة في المستقبل.

## 摘要

近年来，为解决人道主义领域援助工作者实施性剥削、性虐待和性骚扰这一问题已投入了大量资金。然而，这种性行为不端情况仍然是持续存在的复杂挑战，对开展人道主义应援活动的个人和社区（包括对性健康）产生了广泛的影响。本文考虑了有关人道主义环境下的性剥削、性虐待和性骚扰的研究现状，并确定了在加强预防和应对工作所需证据基础方面存在的缺口。首先，我们报告了我们所了解到的性剥削、性虐待和性骚扰情况，包括其对性健康的影响、相关风险因素和宽容的组织文化。然后，我们确定了几个必须解决的关键知识缺口，以便制定更加有效的未来战略和方法来预防和应对性剥削、性虐待和性骚扰。我们讨论了整个系统的知识缺口，例如缺乏关于制定方法以及提高预防和问责机制有效性的证据。我们还探讨了可通过医疗保健项目实施的旨在预防和应对性剥削、性虐待和性骚扰的潜在方案。我们还描述了人口层面的知识缺口，包括犯罪模式和边缘化群体面临的具体挑战。最后，我们针对未来的综合研究和政策议程进行了反思。

## Резюме

В последние годы значительные средства были направлены на борьбу с сексуальной эксплуатацией, надругательствами и домогательствами со стороны работников гуманитарного сектора. Однако подобные сексуальные домогательства остаются постоянной и сложной проблемой, чреватой самыми разнообразными последствиями, в том числе для сексуального здоровья, для людей и общин, принимающих гуманитарные меры. В этой статье рассматривается статус исследований, посвященных сексуальной эксплуатации, надругательствам и домогательствам в гуманитарных контекстах, и выявляются пробелы в доказательной базе, необходимой для укрепления усилий по профилактике и реагированию. Прежде всего в статье представлены сведения о сексуальной эксплуатации, надругательствах и домогательствах, включая их влияние на сексуальное здоровье, а также о факторах риска и о попустительских формах организационной культуры. Затем авторы указывают на некоторые критические пробелы в знаниях, которые необходимо устранить для внедрения более эффективных будущих стратегий и подходов к предотвращению сексуальной эксплуатации, надругательств и домогательств, а также для реагирования на такие явления. Обсуждаются общесистемные пробелы в знаниях, такие как отсутствие фактических данных о подходах к разработке программ и эффективности механизмов профилактики и подотчетности. Кроме того, в статье рассматриваются потенциальные возможности, которые предоставляют программы здравоохранения для предотвращения сексуальной эксплуатации, надругательств и домогательств, а также реагирования на них. Также приведены пробелы в знаниях на уровне населения, в том числе о моделях совершения преступлений и специфических проблемах, с которыми сталкиваются маргинализированные группы. В заключение приведены размышления по поводу будущей комплексной программы исследований и политики.

## Introduction

The World Health Organization (WHO) led the 2018–2020 response to the Ebola virus disease outbreak in the Democratic Republic of the Congo; however, reports emerged in 2020 that WHO personnel had committed widespread sexual exploitation, abuse and harassment (often collectively referred to as “sexual misconduct”) of host communities through well-established sex-for-work schemes.^1^ Although other humanitarian personnel had been implicated in sexual misconduct towards host communities before these accounts, disclosures of perpetration by health sector workers during the Ebola response were particularly disturbing because of the trusted societal role held by WHO staff. Of the 83 allegations identified by a WHO-appointed Independent Commission,^1^ 21 were associated with WHO and of these, nine were substantiated after investigation. 

The events in the Democratic Republic of the Congo highlighted several key issues. First, the Commission’s report demonstrated that, although sexual exploitation and abuse can comprise the same acts as other forms of sexual violence, they had a different meaning for victims and/or survivors because perpetrators had abused their power as paid and trusted personnel of WHO (or other humanitarian organizations); this also means that the organizations hold explicit responsibility for remediating harm caused. The Independent Commission underscored how sexual misconduct by humanitarian personnel affects individuals already rendered most vulnerable by the circumstances that necessitate humanitarian response, namely children, women and those otherwise economically marginalized.

Second, the report highlighted the significant impacts of abuse experienced by victims and/or survivors, including on their sexual health. Sexual health is not only “the absence of disease, dysfunction or infirmity” but also “safe sexual experiences free of coercion, discrimination and violence.”^2^ The consequences of sexual exploitation and abuse include increased exposure to sexually transmitted infections, unintended pregnancies and a long-term disruption to the capacity to enjoy a healthy and satisfying life (including sexual life). Because of subsequent stigma and amplified vulnerability resulting from social and/or familial disconnection, sexual exploitation and abuse exposes victims to increased risks of further abuse and exploitation by both aid and non-aid workers. These consequences align with the sexual health impacts of gender-based violence broadly, which also include negative mental health and gynaecological consequences.^3^^–^^7^ Critically, the revelations of sexual misconduct during the Ebola outbreak response highlighted that sexual misconduct perpetrated by aid workers not only violates the sexual health of victims and/or survivors but undermines trust in services provided by humanitarian organizations, including the subsequently required sexual and reproductive health services.

During the investigations by the Independent Commission, WHO launched a 3-year global strategy^8^ to strengthen its approach to the prevention of and response to all forms of sexual misconduct by its personnel, offering support to all identified victims regardless of the affiliation of the alleged perpetrator. As part of this strategy, WHO prioritized the need to establish a more robust evidence base. This article is part of these efforts, bringing together academic experts on sexual misconduct and WHO staff who contributed to a technical consultation on the state of research on sexual misconduct in humanitarian contexts. In this article, we identify key system-wide and population-specific knowledge gaps that must be addressed for the development of effective prevention and response approaches to the issue of sexual misconduct. We conclude with reflections for future research and policy agendas.

## Understanding sexual misconduct

In 2003, the United Nations (UN) adopted a zero-tolerance policy towards sexual exploitation and abuse by UN personnel and non-UN staff involved in UN peacekeeping and humanitarian missions.^9^ This policy became the foundation of efforts to define, prevent and ensure accountability for such behaviour across peacekeeping and humanitarian sectors, including through its adaptation of the six core principles of the Inter-Agency Standing Committee that apply across the humanitarian sector.^10^ Sexual harassment was later incorporated into the framework of the committee, acknowledging that personnel may also engage in sexual misconduct towards colleagues; this is sometimes referred to as “internal sexual exploitation and abuse,” given that the acts and underpinning power dynamics can be the same as for those perpetrated against aid recipients.^11^

The artificiality of distinctions drawn based on the relationship between perpetrator and victim is conceptually fraught and sometimes distressing to the victim and/or survivor, but reflects the response framework rather than the nature of the act itself. For example, rape perpetrated by a man against his partner is intimate partner violence; by soldier against civilian is conflict-related sexual violence; by aid worker against local child is sexual abuse, covered by organizational sexual exploitation and abuse frameworks; and by an aid worker against a colleague is dealt with using organizational sexual harassment frameworks. The organizational cultures in which sexual exploitation, abuse and harassment arise contradict the expectations of the public and donors regarding the values of aid organizations and the principles underpinning humanitarian work. Critically, these misalignments undermine the trust and security essential for effective humanitarian response.^12^

## Victims and perpetrators 

Key challenges in dealing with sexual misconduct are the wide variety of behaviours it encompasses (only some of which are criminal or violent); its relationship with other forms of gender-based violence, including conflict-related sexual violence; and a lack of comprehensive data to understand prevalence.^13^^,^^14^ The Harmonised Reporting Scheme of the Core Humanitarian Standard Alliance recently published a report summarizing the trends and patterns of reported sexual misconduct incidents received by 30 participating humanitarian organizations between October 2023 and March 2024.^15^ A total of 169 incidents were reported involving sexual misconduct by staff against aid recipients and other staff between October 2023 and March 2024. Of the incidents reported to involve aid recipients (no absolute number provided), 61% reportedly involved allegations of sexual exploitation, 34% of sexual abuse, and 27% of sexual harassment. Note that a single incident may incorporate more than one type of sexual misconduct. Of reported cases perpetrated against fellow staff (no absolute number provided), 87% were classed as sexual harassment. In line with studies on the prevalence of gender-based violence, almost all of reported victims were female, although male survivors likely underreport to a greater degree than females. Among aid recipients, around one third of victims and/or survivors were reported to be younger than 18 years. Reported perpetrators were almost exclusively men (women accounted for 1% of reported incidents), and local staff accounted for around three quarters of reportedly alleged perpetrators. International staff were disproportionately represented given that they comprised less than 10% of staff in most contexts, but accounted for 8% and 5% of all sexual exploitation and sexual abuse allegations, respectively (no absolute numbers provided). Such perpetrators were reported as being middle and senior managers or categorized as other. In around one fifth of the 169 reported incidents, the perpetrator was not identified.^15^

These data provide an indication of trends, although they are not generalizable because of the barriers to reporting that affect the types of incidents reported and by whom; the obstacles to substantiating allegations of sexual exploitation and abuse;^16^ and the fact that they reflect only sexual misconduct allegations within participating organizations. Researchers have estimated gender-based violence is underreported by a factor of 11 to 128.^17^ Furthermore, research suggests that people with diverse sexual orientation, gender identity, gender expression and sex characteristics may experience increased vulnerability because of amplified stigma and reporting obstacles;^11^^,^^18^^,^^19^ however, minimal data exist on other issues that may increase vulnerability, such as language, literacy, and other physical, cultural and social factors. 

Humanitarian spaces, characterized by high levels of material insecurity and fractured community networks, present fertile grounds for the abuse of power and impunity. Because those affected by crises depend on national and international humanitarian personnel to meet basic needs, a power imbalance between aid distributors and recipients is created.^7^^,^^20^^,^^21^ In such contexts, humanitarians and associated personnel (including volunteers, contractors and other intermediaries) may abuse their power by demanding sex in exchange for aid or work.^7^^,^^22^^–^^24^ Displacement has the consequence of fracturing community networks that protect against exploitation and abuse. This context makes women, children and disabled people particularly vulnerable to cycles and economies of exploitation and abuse, especially when relying on men for access to aid distribution points or to transport aid from distribution points back home.^7^^,^^25^^–^^27^ Criminal sex trafficking networks thrive in such contexts, taking advantage of aid distribution systems that do not mitigate such risks.^7^^,^^28^ Where conflict-related sexual violence is widespread, victims and/or survivors are at greater risk of experiencing subsequent sexual exploitation and abuse by humanitarians, especially when stigma, ostracization and unintended pregnancy mean they must care for children born of war alone.^7^^,^^29^ Although women and children are the primary victims, certain factors amplify their vulnerability including disability, economic precarity, physical or familial isolation, and perceived attractiveness.^25^^,^^27^

In the same way in which patterns of perpetration are complex, the impacts of sexual misconduct are severe and wide-reaching. A study on the long-term effects of sexual misconduct by peacekeeping and humanitarian personnel demonstrated damage inflicted at individual and community levels (by compounding human rights abuses, poverty and vulnerabilities of victims and their children); at structural levels (by normalizing and institutionalizing impunity for sexually exploitative and abusive behaviours, and seeding economies of exploitation that outlast international missions); and at operational levels (by diverting resources, causing mistrust of humanitarian workers among local communities, and diminishing the trust humanitarian staff have in their own organizations).^7^ Sexual misconduct also fundamentally undermines sexual health, and increases pressure on gender-based violence services in contexts where they are already oversubscribed and under-resourced, and where stigma for accessing services is high.^30^^–^^32^ Humanitarians are supposed to provide impartial, inclusive and unstigmatized care; when they perpetrate sexual exploitation and abuse, the damage inflicted is multidimensional, amplifying the vulnerabilities that necessitated humanitarian support in the first place, and undermining access to services and support for victims and/or survivors.

We know that sexual misconduct is a social phenomenon whereby individuals who have power and privilege because of their status and wages coerce or violate individuals who are vulnerable because of their material and physical circumstances, often with impunity.^33^ We also know that such behaviour manifests in highly context-specific patterns; the development of policies to prevent and respond to such behaviour depends on an improved understanding of these trends.

## Persistence of sexual misconduct

Historically, sexual misconduct by aid workers has been framed as a policy problem to be solved, with zero cases sometimes held as a marker of success.^20^ These framings have recently shifted at leadership levels; for example, WHO has emphasized that it expects more cases to be reported as prevention and response efforts intensify.^34^ Despite these important shifts, cultural challenges that undermine the prevention of and response to sexual misconduct persist at operational levels, including hostility or retaliation towards staff reporting such behaviour.^20^ Victims, especially local aid workers and aid recipients, are unlikely to report sexual misconduct if they fear community or organizational retaliation, or loss of aid. There is often also an expectation that humanitarians look after themselves: reporting their own experiences of sexual misconduct at the hands of colleagues may be seen to indicate that they lack professional capacity to protect themselves and manage their interactions with colleagues. Such judgements are more likely to be made of aid workers with racialized backgrounds, who often already face discrimination about their professional capacity.^11^ Although there are indications that increasing equity and inclusion can help address these cultural challenges, further research is necessary to better understand these mechanisms for effective policy.

The short-term nature of humanitarian deployments sustains an exceptionality mindset among international personnel, cultivating a permissive working environment and accountability vacuum.^35^ Although dedicated and stable leadership teams can positively influence organizational culture, this positive influence is undermined by institutional hierarchies where leaders and managers are insulated from average humanitarian staff with high contract turnover. Security training and manuals for expatriate humanitarians frame threats of sexual violence as emanating from the field rather than from colleagues or the aid sector itself,^11^ which obscures potential dangers from colleagues and seemingly safe spaces not in the field, such as offices, compounds and headquarters.^11^^,^^36^ This framing compounds an unrealistic, racialized and gendered impression that profiles local non-white men as having the highest capacity for sexual violence, and expatriate white women at highest risk of being victimized.^11^ Even when sexual misconduct is reported, if perpetrator and victim profiles do not fit these expectations then reports may not be taken seriously or may be characterized as the behaviour of rule-breaking outliers. Such impressions prevent sexual misconduct being understood and addressed in the context of its structural causes and permissive environments.^11^

The historical colonial and patriarchal attitudes of the humanitarian sector have also contributed to exceptionality mindsets that allow expatriate workers to see the field as a high-stress environment characterized by alcohol-fuelled parties and casual sex. These cultures intersect with low levels of acceptance of organizational rules and recognition of power differentials among aid workers themselves.^37^^,^^38^ Indeed, data received from new collective reference-checking mechanisms that flag known perpetrators suggest that individuals facing substantiated allegations feel confident about applying for other positions in the sector.^39^ More research on how organizational factors enable sexual misconduct and facilitate impunity is required.

## System-wide knowledge gaps

Most academic research on sexual exploitation, abuse and harassment by aid workers derives from peacekeeping contexts, and focuses qualitatively on the experiences of victims and the factors that give rise to both patterns and individual instances of sexual misconduct; humanitarian sector publications emphasize quantitative data, which is compromised by under-reporting.^15^^,^^17^ Rigorous, systematic and participatory research into risks, mitigation strategies, and challenges to response and accountability efforts in humanitarian contexts is vital for effective policy and practice. Research into how aid programme design affects the risks of sexual exploitation and abuse is also limited;^40^ Empowered Aid is a notable exception.^41^ Most prevention and response approaches have not been evaluated for how and why certain activities achieve impact. A 2021 sector-wide review found that organizations were not monitoring the effectiveness of prevention and response tools or approaches, and therefore did not have evidence to advocate that these be routinely resourced and applied through aid programming.^33^ Such an evidence base can leverage best practice from areas such as sexual and reproductive health and rights, protection, gender and cash programming.

Focusing primarily on individual reports of sexual misconduct means that opportunities to respond to the structural and contextual factors that enable such behaviour may be missed.^13^^,^^20^ Accountability efforts must be accompanied by research into contextually grounded approaches to mitigate sexual misconduct in locally appropriate ways, proactively addressing high-risk settings rather than waiting for reports of abuse.^25^^,^^42^^,^^43^ Research on the roles of individual organizations in creating conducive contexts for abuse and impunity is vital.

The sexual health impacts of sexual misconduct raise the question of how health-care responses could be centred in prevention and response efforts. Health facilities and staff serve as entry points for gender-based violence victims and/or survivors, with packages of services to address physiological and psychological impacts of sexual violence (including novel modalities such as self-care interventions).^44^ These approaches could constitute the response of a health system to sexual exploitation and abuse in humanitarian settings, but these issues are not yet a cross-cutting consideration in service responses. Establishing trust, offering flexible service options and ensuring continuum of care is known to be crucial for effective gender-based violence responses.^45^^–^^48^ Understanding how these methods can be used for victims and/or survivors of sexual misconduct, for whom the aid sector is obliged to provide targeted and holistic support, as well as addressing specific barriers to accessing care, support and justice, is critical. Research exploring how health workers in humanitarian contexts might act as proactive prevention and response agents would be valuable for the integration of service delivery with prevention of and response to sexual misconduct.

## Population-based knowledge gaps

There remains minimal empirical research on perpetration patterns and emerging trends in different contexts; however, such knowledge is critical for locally grounded policy development and programme approaches. Specific types of perpetration (e.g. child abuse, rape, sexual extortion, trafficking, transactional sex between adults, consensual relationships that reflect abuses of power) require different types of prevention, response and accountability mechanisms, the latter of which have been shown to be more suited to some types of sexual exploitation and abuse (especially criminal acts) than others (especially exploitative sex involving adults).^13^ In particular, research tracing the complex interactions of coercion and consent in transactional sex is essential to ensuring that all aid workers – especially those involved in reporting, investigation and disciplinary decisions – have the appropriate knowledge to identify and act on cases of sexual exploitation.^38^^,^^49^

Further research is required on population-specific vulnerabilities to sexual exploitation, abuse and harassment. Although we know marginalized groups are more vulnerable, the different dynamics of their vulnerability require investigation for effective prevention efforts and can yield insight into persistent culture challenges. For example, the humanitarian sector has not adequately addressed the dangers facing aid workers who are part of sexual and gender minority groups,^37^ and the consequences of being openly lesbian, gay, bisexual or transgender in humanitarian spaces can be grave.^50^ In documented incidents of “corrective rape” by colleagues, local homosexual staff reported being raped and “told to keep quiet because otherwise they can be stoned to death”;^18^ others reported the use of blackmail to “pressure victims to do what they told them to do, or face having their sexual identity revealed to their anti-gay colleagues” in locations where homosexuality is criminalized.^18^ Reporting mechanisms do not yet address these dynamics, and research is required to understand the particular vulnerabilities of diverse aid workers.

This issue provides insight into organizational cultures that enable perpetrators of sexual misconduct to act with impunity by instrumentalizing cultures that render victims of diverse sexual orientation, gender identity, gender expression and sex characteristics silent and invisible.^18^ We know equally little about the links between perpetration against colleagues and host communities, and the scope and impacts of such behaviour against aid beneficiaries of diverse sexual orientation, gender identity, gender expression and sex characteristics. There are similarly large population-specific research gaps regarding men and boys, people living with disabilities, elderly women and unaccompanied adolescents.

## Conclusion

Our understanding of sexual exploitation, abuse and harassment perpetrated by aid workers is derived from a less-than-systematic knowledge base, and quantitative data will always be compromised by underreporting. It is therefore vital to employ methods that can illuminate its patterns, dynamics and effects across the affected diverse groups, as well as the challenges these dynamics pose to prevention, response and accountability. Robust, qualitatively grounded research – especially when coupled with data on the risks of sexual exploitation, abuse and harassment as well as mitigation measures derived from ongoing monitoring and evaluation ­– is essential. Equally critical is work that systematically evaluates the impact of specific prevention and response approaches to generate best-practice guidance. Broadening the focus to include approaches from related programming areas (e.g. sexual and reproductive health and rights) can ensure that prevention and response approaches are built on cross-sectoral strengths. Evidence-informed decision-making is essential for making the best use of available resources. Since structural and cultural change will take time, demonstrating the effectiveness of approaches is critical in addressing sexual misconduct by aid workers.
